# To the Editors of the Medical and Physical Journal

**Published:** 1801-02

**Authors:** James Hume Spry


					[ i?7 ]
To the Editors of the Medical and Phyjical Journal.
Gentlemen,
X Hope you will excufe the liberty I am going to take, by
pointing out to you what appears to me an error in your truly
valuable Mifcellany; as fcientific men, I think you will readily
excufe it, and if I am not very much miftaken, will coincide
with me in thinking, that the mode of arrangement adopted
by fome of your correfpondents, is not only irregular, but
unworthy of learned men. I allude to the arrangement of
difeafes in the different diftricls in London. What can be
more difcordant to the eye, or to the ideas of a man who ad-
mires his profeflion, and is a lover of fcience, and who is ac-
quainted with the great accuracy of the celebrated Dr. Cullen
in his Synopfis Nofologiae Adethodicae, as well as the Nofo-
logifts prior to his time, and particularly Sauvages, than to
fee a catalogue of difeafes put together without method, with
half Latin and the other half Englifh names ? Surely, it would
be as eafy to adopt technical terms entirely, or quite reje?t
them in favour of our native language. I confefs that, in my
opinion, technical terms ought folely to be ufed; and I am very
certain that if the method of arrangement was more fyftematic,
it would conduce much to the knowledge of difeafe, indepen-
dant of fhewing the number and quality of difeafes, which
happen within certain ftated periods, in any particular diftrift
of the metropolis, or any other place. Suppofe, for inftance,
Dr. Cullen's method of clarification was chofen, each difeafe
ought to be placed on the lift as it ftands in his catalogue; and
then the medical ftudent, or the juniors in the profeffion, would
fee at one view the nature of each difeafe, and the affinity
they bear to each other.
Classis.
Or do.
No. Genus. Species. Varietas. No.
11 Pyrexia
a Neurofes
3 Cachexias:
4' Locales
xlFtbres
2 jPhlegmafise
3 (Exanthemata
4 iHasmorrbagia
1 jComata
a |IntumefcentitE
Impetegines
Dyiefthefias
Dyfcinefirs
Epifchefes
Dialyfes
1 jTertiana
4!Synocha
51T yphus
101 Cynanche
| Ditto
aC Variola
37 llaemoptyfis
42 Apopkxia
7 51 Anal area
S 5; Syphilis
97 iParacufis
1 iaiParaphwnia ,
127' Amer.orrhcea'
149 'Pfbra
Petechialis
Toniiilaris
Maligna
Difcreta
Phthifica
Sanguinea
Debilium
Rauca
Emanficnis
P3
The
lo8 Dr. Harrison, sn Vaccine Inoculation.
The above table will illuftrate my meaning. The utility
of fuch a table muft be evident to every thinking perfon ; to
the ftudent it would be very valuable, as it would enable him
to fee at one view the nature of any difeafe; and his mind
would be more familiar with, the terms 'of his profefiion; not
to mention, that it would make him ftudy the works of the
immortal Cullen, and thofe of other nofologifts, at prefent too
much neglected. When I firft commenced the ftudy of me-
dicine, I found a table of this kind' of the greateft ufe to me;
it very much aflifted me in acquiring a proper knowledge of
Dr. Cullers Nofologia Methodica; the terms became familiar
to me, and when I was at a lofs for the definition of any par-
ticular term, by looking at my table I immediately faw the
claf's and order, as well as the genus, and from the number of
the genus which was prefixed to each, I made the reference to
the Nofology for an explanation.
Thus, gentlemen, I have freely delivered' my lentiments
upon a fubje6t, which I think requires amendment; and if you
think the above remarks may be of any fervice to our junior
brethren, you are at liberty to infert this paper in your Journal.
I am, &c?
JAMES HUME SPRY*

				

